# Zinc Finger Proteins in Neuro-Related Diseases Progression

**DOI:** 10.3389/fnins.2021.760567

**Published:** 2021-11-18

**Authors:** Siyuan Bu, Yihan Lv, Yusheng Liu, Sen Qiao, Hongmei Wang

**Affiliations:** ^1^Department of Pharmacology, School of Medicine, Southeast University, Nanjing, China; ^2^Department of Pharmacology, Center for Molecular Signaling (PZMS), School of Medicine, Saarland University, Homburg, Germany

**Keywords:** Alzheimer’s disease, ischemic stroke, zinc finger proteins, neuro-related diseases, schizophrenia, epilepsy, autism spectrum disorder

## Abstract

Zinc finger proteins (ZNF) are among the most abundant proteins in eukaryotic genomes. It contains several zinc finger domains that can selectively bind to certain DNA or RNA and associate with proteins, therefore, ZNF can regulate gene expression at the transcriptional and translational levels. In terms of neurological diseases, numerous studies have shown that many ZNF are associated with neurological diseases. The purpose of this review is to summarize the types and roles of ZNF in neuropsychiatric disorders. We will describe the structure and classification of ZNF, then focus on the pathophysiological role of ZNF in neuro-related diseases and summarize the mechanism of action of ZNF in neuro-related diseases.

## The Definition, Structure, and Classification of Zinc Finger Proteins

The first zinc finger protein was discovered as a transcription factor in 1985 by Alan Klug of the Laboratory of Molecular Biology in Cambridge, England. Zinc finger proteins (ZNF) can form transcription initiation complexes that bind to DNA-specific regions and mediate transcription. ZNF mainly rely on Zn^2+^ to form a stable structure similar to “finger” (Berg and Shi, 1996). Zn^2+^ binds with different amounts of Cys and His to form different types of ZNF. ZNF are classified in two different ways. Classic zinc finger—The C2H2 zinc finger protein includes two conserved cysteines and two conserved histidine residues combined with zinc ion which can form two β sheets and an α helix. The HUGO Gene Nomenclature Committee (HGNC) divides non-classical types of zinc-finger into 30 types according to their different amounts of Cys and His in 2015 (Gray et al., 2015).

Another classification of ZNF is based on the different spatial structures of Cys and His around Zn^2+^. The ZNF discovered so far can be classified into eight types according to the spatial structure of their zinc finger binding sites including Cys2His2 (C2H2) like, Gag knuckle, Treble clef, zinc ribbon, Zn2/Cys6, TAZ2 domain like, short zinc binding loops, and Metallothionein (Krishna et al., 2003). According to the structure of zinc finger protein, specific binding target structure can be selected, so the function of zinc finger protein is also varied. Most of the classic ZNF, C2H2, binds to DNA, and some ZNF bind to RNA or proteins. ZNF play an important role in cell differentiation, embryo development and other life processes. Among the more than 30 ZNF that have been found, the ZNF that play an important role in the brain are mainly C2H2-type, MYM-type, Matrin-type, Zinc fingers CCCH-type (ZC3H) and Ring ZFP, as detailed in the Table 1 below.

**TABLE 1:** Types of Zinc finger proteins (ZNF).

**Zinc-protein Types**	**Important members**	**Physiological functions in brain**	**References**
C2H2 type (Kruppel-like factor 4)	Teashirt family zinc finger 1, EHZF/ZNF521, KLF4, KLF11, MZF1, ZNF667, PLZF, ZNF208, GLIS3, ZFX, GLI3, ZEB1, ZEB2, ZBTB16, ZKSCAN3, Zfp189, ZNF746, ZNF423, ZNF711, ZNF462, ZNF292, ZNF526, ZBTB5, ZNF804A	a. Bind to DNA specifically and promote DNA transcription. b. The proper folding of some C2H2 ZFs is important to the interaction of proteins.	Frankel et al., 1987; Wolfe et al., 2000; Schefe et al., 2006; Brayer et al., 2008; Town et al., 2009; Fan et al., 2012; Shi et al., 2013; Yuan et al., 2013; Xu et al., 2016; Yu et al., 2017; Brahmachari et al., 2019; Kruszka et al., 2019; Lorsch et al., 2019; Ohkubo et al., 2019; Zhang et al., 2019; Choi et al., 2020; Deshpande et al., 2020; Hu et al., 2020; Xiao et al., 2020; Dentici et al., 2021; Poeta et al., 2021
CCCH-type (ZC3H)	MCPIP1, ZC3H14, Zn72D, ZC3H4	a. Interact with RNA to control many steps of RNA metabolism. b. Including mRNA splicing, transport and translation. c. Play an antiviral and immuno-homeostasis role by regulating RNA metabolism in viruses and activating immune cells and affecting the production of cytokines.	Liang et al., 2011; Kelly et al., 2012; Hajikhezri et al., 2020; Estell et al., 2021
Cys3HisCys4 (Ring ZFP)	ZNF179, KRAB-ZFP	a. As an E3 ubiquitination ligase. b. Regulate the ubiquitination and degradation of target proteins to regulate a variety of physiological processes in cells. c. Used as a targeted inhibitor of MDM2 to activate P53 and induce apoptosis in cancer cells.	Haupt et al., 1997; Su et al., 2016; Liu et al., 2018
MYM-type	Zinc finger MYM-type protein 3	a. ZMYM3 as a master regulator is related to AD and cognitive impairment in humans. b. ZMYM3 is a component of histone deacetylase function through modifying chromatin structure to keep genes silent.	Afshar et al., 2020
Matrin-type	CIZ1	a. CIZ1 induces the cytoplasmic export of CDKN1A (p21 CIP1), blocking the onset of S phase and leading to disruption of cell function in AD. b. The protein encoded by CIZ1 is a zinc finger DNA binding protein that interacts with CIP1, part of a complex with cyclin E.	Dahmcke et al., 2008
PHD-type	BAZ2B	a. BAZ2B plays a role in transcriptional regulation *via* interaction with ISWI b. BAZ2B contributes to the normal neurodevelopment.	Jones et al., 2000; Scott et al., 2020
Metallothionein protein	MT-I, MT-II, MT-III	a. Metallothionein proteins can bind to brain zinc. b. Metallothioneins-III plays a neuroprotective role to protect neuronal cells against reactive oxygen species (ROS)	Sohn et al., 2012

## Biological Function of Zinc Finger Protein in the Brain

In many tissues of the human body, including the brain, ZNF play a role in regulating the specific expression of the human genome as transcription factors by specifically binding to DNA and RNA (Farmiloe et al., 2020). Post-transcriptional control can occur at each step of RNA metabolism, including splicing, capping, polyadenylation, export, localization, translation, and decay. ZNF are generally thought of as DNA-binding transcription factors. Many studies have shown that the main function of ZNF in the brain is to promote the development of different parts of the brain and the differentiation of neural stem cells. For example, the C2H2-type zinc finger protein, EHZF/ZNF521, was found to have a possible regulatory effect on the development of the cerebellum in the *in situ* hybridization data of the brain gene expression map (Magdaleno et al., 2006). It has been shown that ZNF521 may regulate differentiation in different regions of the brain through their interaction with EBF (Garcia-Dominguez et al., 2003; Bond et al., 2008). Another C2H2-type zinc finger protein, Teashirt family zinc finger 1, was found to be enriched in the striatum. The Teashirt family zinc finger 1 (Tshz1) adrives negative reinforcement and is essential for aversive learning. The Tshz1 neurons cause aversion, movement suppression, and negative reinforcement once activated, and they receive a distinct set of synaptic inputs (Xiao et al., 2020). It has been hypothesized that the RING type zinc finger proteins KRAB-ZFPs can act together with TRIM28 to repress the expression of transposable elements thus having a positive effect on the evolution of the brain genome toward complexity (Grassi et al., 2019; Farmiloe et al., 2020). Another gene, the loss of ZC3H14, affects the post-transcriptional RNA regulatory mechanisms to compartmentalize gene expression in time and space (Cooke et al., 1987). Zinc finger protein at 72D (Zn72D) as a regulator of editing levels at a majority of editing sites in the brain. Zn72D is necessary to maintain proper neuromuscular junction architecture and fly mobility (Sapiro et al., 2020). ZNF423 (C2H2 type), ZC3H4 are also associated with neurodevelopment (Deshpande et al., 2020; Estell et al., 2021; Su et al., 2021). ZBTB5 (zinc finger and BTB domain-containing 5) from C2H2 type subfamily is a proto-oncogene that stimulates cell proliferation (Choi et al., 2020).

## Role of Zinc Finger Proteins in Neuro-Related Diseases

The most common studies on neuro-related diseases include Alzheimer’s disease (AD), stroke, schizophrenia, depression, anxiety, trauma, epilepsy, autism and so on. ZNF are mainly studied in AD, stroke, schizophrenia, epilepsy and autism. We will introduce the mechanism of zinc finger protein in these neuro-related diseases from the following diseases.

### Alzheimer

Alzheimer’s disease is a degenerative disease of the central nervous system characterized by progressive cognitive dysfunction, and often occurs in the elderly (Soria Lopez et al., 2019). The current confirmed pathogenic factors of AD include the formation of senile plaques induced by abnormal amyloid-β (Aβ) deposition and the neurofibrillary tangles or dystrophic neuritis induced by tau accumulation (Shinohara et al., 2014; Soria Lopez et al., 2019). C2H2-type, MYM-type, ZC3H14, and Matrin-type of ZNF are reported in AD.

**FIGURE 1:**
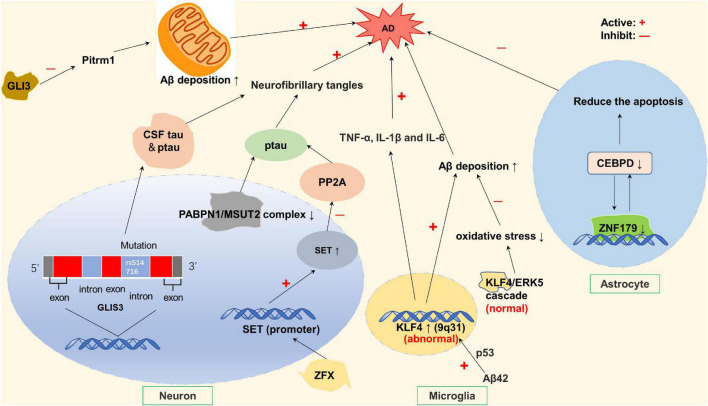
Zinc finger proteins (ZNF) associated with Alzheimer’s disease (AD). Rs514716 in GLIS3 is associated with cerebrospinal fluid (CSF) tau and ptau levels, which is a risk gene for AD. ZFX can activate SE translocation (SET) promoter and inhibit PP2A phosphatase, resulting in tau hyperphosphorylation. Loss of PABPN1/MSUT2 complex can aggravate the severity of AD. The oligomer AB42 increased the expression of KLF4, and the overexpression of KLF4 increased the increase of TNF-α, IL-1β, and IL-6, thereby exacerbating AD. However, under normal conditions, ERK5/KLF4 cascade reaction can avoid oxidative stress-induced neuronal death. GLI3 expression inhibited the activity of endometrial peptidase and reduced the degradation of Aβ in mitochondria. CCAAT/enhancer binding protein delta (CEBPD) is an upstream regulator of the ZNF179 gene in astrocytes. CEBPD regulates the transcription of ZNF179 by directly binding to the promoter region of ZNF179 and exerts an anti-apoptotic effect on astrocytes.

Studies show that GLIS family zinc finger 3 (GLIS3) and ZFX belonging to C2H2 family, MSUT2 (ZC3H14) belonging to CCCH-type family influence the accumulation of tau proteins to affect the neurofibrillary tangles. GLIS3 is a zinc finger protein related to tau protein, and is highly expressed in islet β cells, the gene which it corresponds to is a risk gene for Type 1 and Type 2 diabetes, glaucoma and AD endophenotype (Scoville et al., 2020). The development of AD is associated with mutations in the DNA intron that codes for GLIS3: A genome-wide association study of cerebrospinal fluid (CSF) tau/ptau levels has shown that mutations in rs514716 in DNA introns encoding GLIS3 were more likely to lead to endomorphic AD (Cruchaga et al., 2013; Calderari et al., 2018). And this is just one of the AD risk genes, in addition to rs9877502 located between GEMC1 and OSTN, and rs6922617 within the TREM gene cluster. These rare mutations promote accumulation of pathological tau protein (Cruchaga et al., 2013). ZFX is associated with pathological tau protein formation (hyperphosphorylation), resulting in the formation of neurofibrillary tangles typical of AD (Xu et al., 2016). ZFX can activate SET as its upstream regulator. For SET transcript 2 promoter, ZFX-mediated transactivation depends on the proximal promoter region containing four ZFX-binding sites, and the translated SET protein can inhibit protein phosphatase 2A (PP2A) (Xu et al., 2016). Inhibition of PP2A promoted abnormal phosphorylation of tau protein (Arif et al., 2014; Janghorban et al., 2014). This is similar to the way in which SET is overexpressed in over half of breast cancers, resulting in inhibition of PP2A and subsequent activation of the transcription factor c-MYC by phosphorylation at serine 62 (S62) (Janghorban et al., 2014). Meanwhile, the mammalian suppressor of tauopathy 2 protein (MSUT2), a poly(A) RNA binding protein, functions to bind poly adenosine [poly(A)] tails of mRNA through its C-terminal CCCH type zinc finger domains, which leading to the formation of pathological tau protein (Baker et al., 2020). Studies have shown that MSUT2 knockout has anti-inflammatory and neuroprotective effects in a mouse model. The flowchart is shown in Figure 1. Loss of function of MSUT2 can reduce the formation of tau protein and protect the loss of neurons, but the molecular mechanism is still unclear. It can only be said that this may be related to the joint action of MSUT2 and its antagonistic canonical nuclear poly(A) binding protein PABPN1 (Wheeler et al., 2019; Baker et al., 2020).

In addition to neurofibrillary tangles in neural cells formed by tau hyperphosphorylation, there is extracellular senile plaque formation resulting from amyloid beta deposition in characteristic pathological changes of AD (Soria Lopez et al., 2019). Studies show that KLF4 and GLI3 belonging to C2H2 subfamily, are related to abnormal amyloid-β (Aβ) deposition. Krüppel-like factor 4 (KLF4), a dual-functioning transcription factor in the zinc finger transcription factor family, its expression positively correlated with Aβ42-induced neuroinflammation (Li et al., 2017). Normally, there is a balance between KLF4 and ERK5, antagonizing each other, KLF4 protects neurons from oxidative stress-induced apoptosis through interacting with ERK5 to maintain genomic stability (Griffin and Barger, 2010; Cai et al., 2011; Li et al., 2017), and Aβ deposition is normal in the equilibrium state, maintaining neurons against oxidative stress-induced apoptosis (Cheng et al., 2018). However, when KLF4 is excessive, Aβ deposition is abnormal, possibly triggering AD. Oligomeric Aβ42 increases KLF4 expression, which is mediated by P53 activation by increased phosphorylation at ser15, then leads to abnormal amyloid-β (Aβ) deposition in microglial BV2 cells (Kaushik et al., 2013; Cheng et al., 2018). Another protein associated with Aβ deposition is GLI3. Expression of GLI3 can inhibit Pitrm1 (pitrilysin metallopeptidase) activity, which is a metalloendopeptidase and is able to degrade amyloid-β when it accumulates in mitochondria. This leads to decreased amyloid degradation, which obviously promotes AD development (Falkevall et al., 2006; Alikhani et al., 2011; Sekar et al., 2015). Moreover, GLI3 expression is inhibited by presenilin 1 (PSEN1), although this is shown temporarily only in-Xenopus embryos (Paganelli et al., 2001). However, PSEN1 is part of the γ-secretase complex and is involved in the production of Aβ amyloid, which leads to reduced neuronal differentiation, which may be involved in the occurrence of familial early-onset AD (Bateman et al., 2011; Hernandez-Sapiens et al., 2022; Figure 1).

Although not directly associated with Aβ deposition, ZNF179 plays a protective role in AD by regulating the CCAAT/enhancer binding protein delta (CEBPD), which is a transcription factor found in activated astrocytes that surround β-amyloid plaques. CEBPD can enhance the anti-apoptotic ability of astrocytes (Figure 1; Wang et al., 2015; Cai et al., 2017). Similarly, studies have shown that MT-I (Metallothionein-I) and MT-II (Metallothionein-II) are overexpressed in astrocytes and microglia surrounding amyloid plaques (Hidalgo et al., 2006; Kim et al., 2012). MT (Metallothionein) expression is induced by AD-associated inflammatory mediators, thereby playing a neuroprotective role (Hidalgo et al., 2001; Reddy, 2006; Juarez-Rebollar et al., 2017). MTI can protect astrocytes by attenuating Aβ neurotoxicity or inhibiting Aβ-induced microglia activation (Valko et al., 2007). It has also been reported that MT-2A (Metallothionein-2A) can prevent the accumulation of Aβ40 and Aβ42 to reduce neurotoxicity (Santos et al., 2012). In addition, MT-III (Metallothionein-III) expression is reduced in AD mouse model, and it has a protective effect by eliminating toxic aggregates of Aβ peptides (Yu et al., 2001; Irie and Keung, 2003). In addition to being associated with the pathological changes typical of AD, ZNF also regulate the expression of AD-related genes. ZMYM3 (DXS6673E or ZNF261) and CIZ1 (Matrin-type) affect the expression of AD-related genes in terms of molecular structure. The human X-linked zinc finger MYM-type protein 3 (ZMYM3) is one of the three master regulators (MRs) related to late-onset AD, which is responsible for disease progression (Aubry et al., 2015). ZMYM3 contains the longest GA-STR which gives it an advantage in the selective expression of human genes. Alleles of the GA-STR complex were found to be associated with late-onset neurocognitive disorder (NCD), schizophrenia and bipolar disorder (Alizadeh et al., 2019; Afshar et al., 2020). ZMYM3 can regulate the transcriptional signal of AD, and severe AD is often accompanied by low level of ZMYM3 expression in the brain nucleus (Alizadeh et al., 2019; Afshar et al., 2020). Cdkn1A-interacting zinc finger protein 1 (CIZ1) can bind to DNA and regulate DNA replication, entering to S phase by inducing the cytoplasmic export of CDKN1A (p21 CIP1), which is a major factor in blocking the onset of S phase (Dahmcke et al., 2008; Liu et al., 2016). The inappropriate re-entry of post-mitotic neurons into the cell cycle may lead to disruption of cell function in AD (Liu et al., 2016).

In addition, it has been shown that Myc-interacting zinc-finger protein 1 (Miz1) from C2H2 type may block normal signaling at synapses based on post-translational modification, leading to AD, and may also obstruct the coupling of small ubiquitin-like modifier (SUMO) and reduce the activity of SUMO ligase, leading to synaptic dysfunction (Wolf et al., 2013; Lee et al., 2015; Liu et al., 2017).

### Ischemic Stroke

Stroke is classified into hemorrhagic stroke and ischemic stroke. Ischemia, hypoxia, and inflammation in the brain are the typical pathophysiological mechanisms of ischemic stroke. Blood brain barrier (BBB), tight junctions (TJs) and astrocytes are closely related to ischemic stroke (Nico and Ribatti, 2012).

**FIGURE 2:**
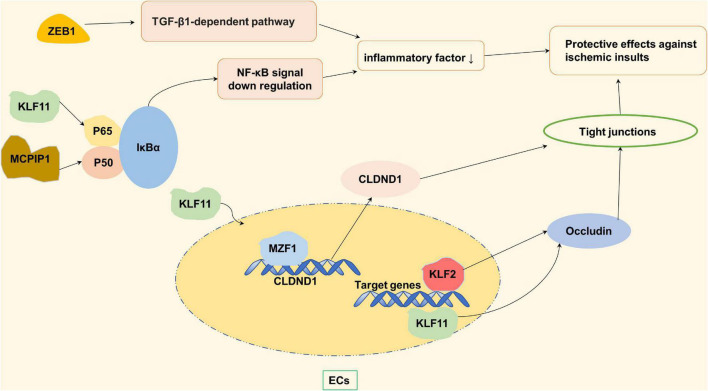
Zinc finger proteins (ZNF) associated with protective effects against ischemic insults. KLF2 and KLF11 maintain the function of blood brain barrier (BBB) by regulating the occludin in the vascular endothelial cells. KLF11 and MCPIP-1 reduce the level of inflammatory factors by negatively regulating NF-κB pathway, thus protecting BBB. MZFI promotes TJs synthesis by increasing the mRNA and protein expression levels of its potential binding site CLDND1, and regulates vascular permeability.

KLF2, KLF11, MZF1, and MCPIP1 play a protective role in stroke by protecting BBB. Krüppel-like factor 2 (KLF2) is expressed within the cerebrovascular endothelium. KLF2 regulates several key BBB tight junction factors, most notably occludin, thereby serving as a positive mediator of BBB function, reducing compound in the blood into the cerebral vessels and reducing the risk of stroke (Shi et al., 2013). After an ischemic stroke occurs, KLF11 functions at microvascular endothelial cells to promote tight junction proteins activities and reduce the expression of pro-inflammatory factors—IL-6 (Fan et al., 2012), and maintain the structural and functional integrity of BBB (Neve et al., 2005; Fernandez-Zapico et al., 2009; Zhang et al., 2020), thus providing brain protection in ischemic stroke. Conversely, KLF11 gene deficiency significantly aggravated ischemia-induced BBB destruction (Zhang et al., 2020). The detailed molecular mechanism is shown in Figure 2.

Tight junctions are related to cerebral vascular permeability. Increased cerebrovascular permeability can lead to an increased risk of stroke (Nico and Ribatti, 2012). Claudin domain containing 1 (CLDND1) is a potential binding site of the myeloid zinc finger 1 (MZF1). In pathological conditions, the down-regulation of whose gene can affect TJs, which can lead to increased permeability of the brain’s vascular endothelium. Under normal circumstances, MZFI promotes the synthesis of TJs by increasing the mRNA and protein expression levels of CLDND1, and better regulates vascular permeability, thus exerting its protective effect on the cerebrovascular system (Davis et al., 2019; Shima et al., 2021). It is worth mentioning that the protective effect of leukemia inhibitory factor (LIF) on brain nerves also depends on the signaling of MZF-1 (Davis et al., 2019). In addition to increased vascular permeability, a decrease in the number of blood vessels in the brain also contributes to an increased risk of stroke. Vascular endothelial zinc finger 1 (VEZF1) promotes ischemic brain injury by targeting miR-191 to inhibit angiogenesis (Du et al., 2019).

Furthermore, Inflammation after ischemic stroke can induce more apoptosis of nerve cells and cause irreversible brain damage. Monocyte chemoattractant protein-1 (MCP-1) binding to its receptor CCR2 induces a novel zinc finger protein, named MCP-1-induced protein 1 (MCPIP1, ZC3H12A) (Liang et al., 2010), which negatively regulates the inflammatory response by inhibiting the NF-κB signaling pathway (Liang et al., 2010; Strecker et al., 2011). MCPIP1 also functions as a RNase promoting inflammatory mRNA degradation (Musson et al., 2020). In the absence of MCPIP1, the expression of matrix metalloproteinase (which destroys extracellular matrix and destroys the BBB) and the expression of Claudin-5 (integrin of TJs) is decreased, which aggravates BBB destruction (Jin et al., 2019). In addition to MCPIP1-mediated inflammatory responses, ZEB1 (C2H2 type) in microglia after ischemic stroke can reduce neutrophils infiltration into ischemic brain by reducing the production of C-X-C motif ligand 1 (CXCL1) in astrocytes (CXCL1 is a chemokine that mainly absorbed neutrophil), thereby alleviating nerve injury (Li et al., 2018).

**FIGURE 3:**
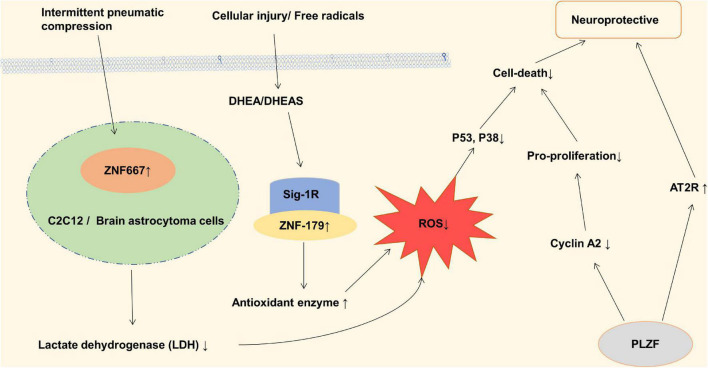
Zinc finger proteins (ZNF) associated with neuroprotective in ischemic stroke. Dehydroepiandrosterone (DHEA) and its sulfated analog (DHEAS) in adrenal and cerebral glands increase antioxidant enzyme levels, inhibit oxidative stress, and reduce nerve cell death through Sig-1R and ZNF-179 pathway. ZNF667 plays a role as a transcription suppressor in IPC-induced neuroprotection, thereby protecting C2C12 and brain astrocytoma cells from oxidative stress induced damage. Promyelocytic leukemia zinc finger (PLZF) induces neuroprotective endophoric phenotypes by regulating neuroprotective and neurotoxic target genes, such as AT2R and Cyclin A2.

Stroke is closely related to the accumulation and invasion of reactive oxygen species (ROS). Both ZNF179 (Ring type) and ZNF667 (C2H2 type) play a role in stroke-related oxidative stress (Figure 3). The brain ZNF179 is a downstream target for the regulation of sigma-1 receptor (Sig-1R), which has a protective effect on neurons after the stroke (Kuo et al., 2020). ZNF179 may prevent ROS-induced neurodegenerative and neurotraumatic disease damage, and which may be mediated by the influence of antioxidant enzyme levels (Su et al., 2016; Wu et al., 2018). In the case of ischemia injury and ROS invasion, zinc finger protein667 acts as a transcription suppressor by means of its KRAB domain in the N-terminus. ZNF667 protects brain astrocytoma cells from oxidative stress-induced damage, and plays a role in brain protection after ischemic injury (Yuan et al., 2013). *In vitro* studies have found that transcription factor promyelocytic leukemia zinc finger (PLZF) from C2H2 type has anti-proliferative effects as a suppressor of cyclin A2 and a neuroprotective factor on human neurons. After stroke, PLZF may play a neuroprotective role by inhibiting cyclin A2, reducing neuronal cell activation and continuous death (Schefe et al., 2006; Seidel et al., 2011). In addition, Zinc as an essential nutrient plays an anti-inflammatory and antioxidant role. As a regulator of zinc homeostasis, metallothionein proteins can balance free and bounded zinc ions in human body after a transient cerebral ischemia. After the occurrence of ischemic stroke, inflammatory responses which are induced by macrophages and microglia cells can increase the Intracellular concentration of MT-I and MT-II which can protect against the oxidative stress (Chung et al., 2003; West et al., 2011). Also, after the stroke, the extracellular free zinc accumulates specifically in degenerating neurons, which might be the mechanism of selective neuronal death in stroke (Koh et al., 1996). Metallothionein proteins can bound zinc to reach saturation, therefore might exert protective role to neuron in stroke. Metallothioneins-III plays a neuroprotective role to neuronal cells against ROS (Sohn et al., 2012; Choi et al., 2018).

Some ZNF may influence the incidence of stroke by related genetic variation. Statistical results of clinical cases showed that the polymorphisms rs2188971 and rs7248488 within the gene ZNF208 were associated with a decreased risk of ischemic stroke in a southern Chinese Han population. The variant rs2788972 of zinc finger protein208 increased the susceptibility to ischemic stroke (Yu et al., 2017).

### Schizophrenia

Schizophrenia (SCZ) is a chronic, severe mental disorder that involves an individual’s sensory, emotional, and behavioral abnormalities. Studies have shown that ZNF are associated with schizophrenia. Although at present the pathogenesis of schizophrenia is not very clear, studies have shown that in some patients, schizophrenia is characterized by dopamine (DA) deficiency in the mesocortex and in the mesolimbic dopamine neurons. The high and low activity of dopamine in the brain of schizophrenic patients may exist at the same time, which has implications for the role of dopamine in schizophrenia.

**FIGURE 4:**
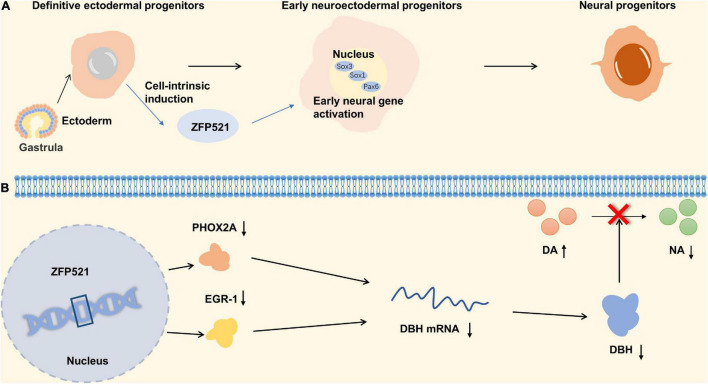
Zinc finger proteins (ZNF) associated with schizophrenia. **(A)** The relationship between ZNF521 and the differentiation of neural progenitors. **(B)** The mechanism of ZNF521 in schizophrenia.

C2H2 type subfamily plays an important role in schizophrenia including ZNF521 and ZEB2. ZNF521, a transcriptional regulation factor with 30-zinc finger protein, acts as a molecular switch for the differentiation of neuronal stem cells and can also promote the proliferation and differentiation of striatal neurons. ZFP521 protein is associated with neuroectodermal specific gene loci (Sox1, Sox3, and Pax6), and can promote neural differentiation by activating early neuroectodermal gene expression. In addition, ZFP521 is co-located with P300 at the neuroectodermal locus, which requires ZFP521 to facilitate early nervous system differentiation (Lobo et al., 2008; Kamiya et al., 2011). The lack of ZFP521 results in a reduced number of granular neurons and an indistinct border of the granular cell layer of the dentate gyrus of hippocampus (Figure 4A; Ohkubo et al., 2014). Studies have shown that decreases in neural stem cell proliferation and adult hippocampal neurogenesis in the dentate gyrus were associated with schizophrenia (Reif et al., 2006; Balu and Lucki, 2009). The results of the behavior test suggested that ZNF521^–/–^ mice have a hyper-locomotive phenotype which is a classical feature of rodent models of schizophrenia and corresponds to the clinical symptoms of patients with schizophrenia (Gainetdinov et al., 2001; Miyakawa et al., 2003). Besides, the locomotor behavior in mice is reported to be dependent on the quantitative balance between DA and NA (Viggiano et al., 2004). Dopamine β-hydroxylase (DBH) is a catecholamine biosynthase that can convert DA to NA, and its level decreases with the upregulation of ZFP521 protein. The expression of ZFP521 inhibited the mRNA levels of PHOX2A and EGR-1 proteins, which are the main transcription factors of DBH, so the expression of ZFP521 inhibited the expression of DBH. Neurotransmitters are regulated by DBH levels in ZNF521^–/–^ mice, which performances for the decrease of DA level and the increase of NA level (Figure 4B; Ohkubo et al., 2019). According to the pathological manifestations and the level of neurotransmitters in the brain, deficiency of ZNF521 can lead to manifestations associated with schizophrenia.

The ZEB2 gene, a key regulator of epithelial differentiation, encodes the zinc finger E-box binding protein (C2H2 type). A case-control study implicated that rs6755392 locus in ZEB2 gene was significantly associated with SCZ. There are indirect protein-protein interactions between proteins encoded by ZEB2 and QPCT genes, and the related pathways also appear to be prominent in the genetic etiology of schizophrenia (Khan et al., 2016). A genome wide association study (GWAS) and comprehensive meta-analysis in a Caucasian population showed that SNP rs12991836, the most recent gene for ZEB2, is a risk locus for SCZ (Ripke et al., 2013).

In the generation of γ-aminobutyric acid GABAergic cortical interneurons, ZEB2 is needed to repress the Nkx2-1 homeobox transcription factor. When ZEB2 is deficient, the differentiation of striatal neurons is affected (McKinsey et al., 2013). GABAergic interneurons play an important role in the synchronization of brain activity in different regions, and abnormalities can lead to psychosis (Keverne, 1999). To sum up, ZEB2 is an important potential target in schizophrenia. The detailed molecular mechanism is shown in Figure 4.

### Epilepsy

**FIGURE 5:**
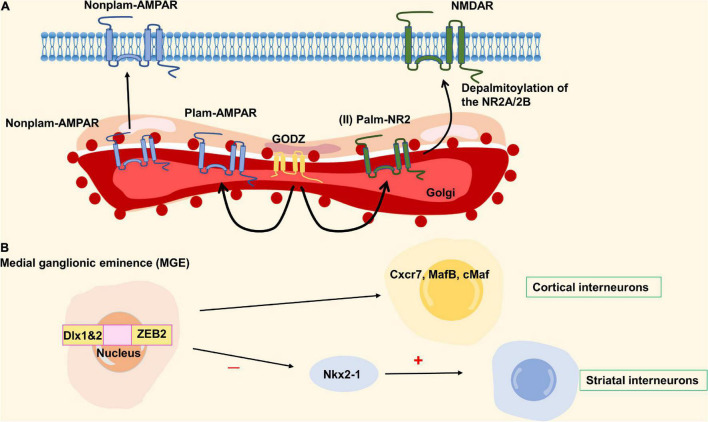
Zinc finger proteins (ZNF) associated with epilepsy. **(A)** The network of GODZ in epilepsy. **(B)** The ZEB2 homobox gene produces cortical interneurons expressing cxcr7, mafb, and cmaf downstream of Dlx1&2. When ZEB2 is deficient, Nkx2-1 is not inhibited, and the cells differentiated into cortical interneurons turn into striatum interneurons. ZEB2 is necessary to generate cortical interneurons, and its mutation can cause epilepsy.

Epilepsy is an acute, recurrent, paroxysmal brain disorder caused by the excessive discharge of neurons in the brain. Golgi-specific DHHC type zinc finger protein (GODZ) is a member of the DHHC protein family that plays an important role in the physiology and pathophysiology of the nervous system, especially in neuronal development and synaptic activity. AMPA receptors, NMDA receptors, and GABA_a_ receptors are known to play important roles in the pathogenesis of epilepsy. Among them, GODZ enhanced the activity of the GABA_a_ receptor that can decrease epileptic seizures. Studies have shown that the disorder of GABA_a_ receptor transport mechanism may lead to the occurrence of epilepsy in GODZ^–/–^ mice (Faheem et al., 2014). The upregulation of AMPA receptors in cell membranes can lead to hyperexcitability of the somatosensory cortex, leading to epilepsy (Kennard et al., 2011). GODZ may exert antiepileptic effects through palmitoylated AMPA receptors by decreasing the number of AMPA receptors on the cell surface (Hayashi et al., 2005; Wang et al., 2018). Another mechanism that causes epilepsy is that GODZ-mediated palmitoylation at the Cys cluster II site of NR2A and NR2B subunits prevents the expression of NMDARs on the neuronal surface (Hayashi et al., 2009). It has been reported that the NMDA receptor was closely related to epileptogenesis (Lau and Zukin, 2007). The detailed molecular mechanism is shown in Figure 5A. The upregulation of NMDA receptors can promote the occurrence of epilepsy, but its specific mechanism is not clear. GODZ may have a protective effect on epilepsy by reducing NMDA receptors on the neuronal surface. In addition, Zfhx1b (Sip1, Zeb2) zinc finger homeobox gene subpallial expression is directly positively regulated by Dlx1&2, one of three parallel transcriptional pathways in the MGE that are required for cortical interneuron development (Long et al., 2009a,b). Also, Zfhx1b is required in the MGE to generate cortical interneurons that express Cxcr7, MafB and cMaf. In its absence, Nkx2-1 expression is not repressed, and cells that ordinarily would become cortical interneurons are transformed toward the NPY/nNos/Sst subtype of striatal GABAergic interneuron. The development disorder of cortical interneurons may cause the clinical symptoms of Mowat-Wilson syndrome, which is characterized by epilepsy (Adam et al., 1993; McKinsey et al., 2013). Therefore, ZEB2 has a certain correlation with epileptic seizures (Figure 5B). Golgi-specific DHHC type zinc finger protein is a member of the DHHC protein family and its enzymatic activity is regulated by fibroblast growth factor or Src kinase-mediated tyrosine phosphorylation. Epilepsy may reduce the protein and mRNA levels of GODZ, indicating a possible role of GODZ in the pathogenesis or the pathophysiology of epilepsy (Wang et al., 2018).

In addition, metallothioneins are low molecular weight proteins with the ability to bind metals. There are four subtypes of metallothioneins (I–IV), which have a high affinity for Zn and maintain its equilibrium and transport (Kimura and Kambe, 2016). Metallothionein has been shown to be associated with epilepsy. Kainic acid (KA) can trigger an inflammatory response in damaged brain regions, suggesting that oxidative stress produced by KA is able to induce seizures (Dalton et al., 1996). Kim et al. (2003) found an increase in the mRNA level of MT-I and MT-II in the brain of rats injected with KA (Juarez-Rebollar et al., 2015). Studies have shown that high levels of MT-I and MT-II can reduce the neuronal death following an induced seizure attack during excitotoxicity. There are also increased levels of oxidative stress, neuronal death, and epileptic seizures after being treated with KA in the MT-I and MT-II knockout mice (Carrasco et al., 2000). On the contrary, MT-I overexpression can reduce inflammation in the hippocampus and delay neurodegeneration (Penkowa et al., 2005). In addition, MT-III prevented KA-induced seizures and reduced neuronal damage in wild-type mice (Erickson et al., 1997).

### Autism Spectrum Disorder

Autism is the representative disease of widespread developmental disorders. Patients often show the barriers of communication with others, intellectual abnormalities and so on. The mechanism of autism is unclear. Both environmental and genetic factors contribute to autism. However, current studies mainly focus on the influence of genes on autism due to the high heritability and familial aggregation of autism (Chaste and Leboyer, 2012). Abnormal signaling pathways, copy number variation and gene variation can lead to increased risk of autism (Sharma et al., 2019; Rahi and Mehan, 2020). ZNF, particularly those from the C2H2 subfamily, have been linked to autism.

Gli from C2H2 type subfamily through sonic hedgehog (Shh) signaling pathway can make the central nervous system develop normally, promote the normal proliferation and differentiation of neurons, and play a neuroprotective role. Shh can bind to the receptor Patched (Ptch), which binds and activates smoothened (SMO), forming a complex that allows Gli to activate and enter the nucleus as a transcription factor to promote the expression of downstream genes (Rahi and Mehan, 2020; Li et al., 2021). Halepoto et al. (2015) demonstrated that children with autism have higher serum levels of Shh than normal children, that is, there is a link between abnormal shh signaling pathway and autism. Under stress conditions, SHH ligands do not bind to the receptor for Patch1, Patch1 still inhibits the SMO and GLI1 is unable to be activated by SMO, resulting in down-regulation of SMO-SHH signaling and abnormal increase in oxidative stress and neuronal inflammation leading to the development of autism (Rahi and Mehan, 2020).

**FIGURE 6:**
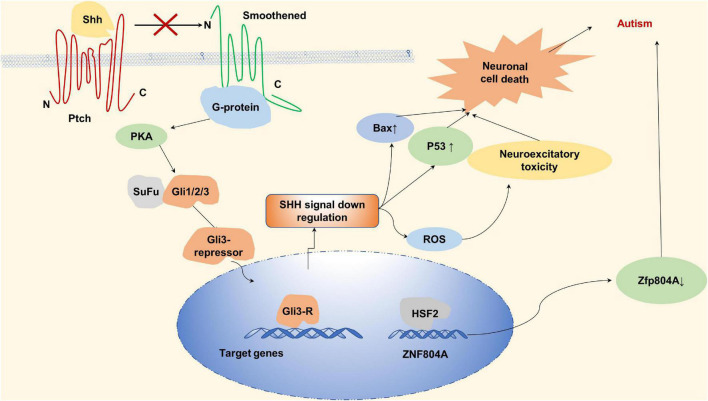
Zinc finger proteins (ZNF) associated with autism. Sonic hedgehog can bind to the receptor Patched (Ptch), Ptch binds and activates Smoothened (SMO) to form complexes that activate Gli and promote downstream gene expression by entering the nucleus as a transcription factor. In the absence of sonic hedgehog, sonic hedgehog (SHH) ligand does not bind to the receptor of Patch1 under stress conditions, PTCH inhibits SMO, and Gli proteins are phosphorylated by protein kinase A (PKA) to form A gli3-repressor that repress transcription of target genes. Downregulation of SMO-SHH signal, abnormal increase of oxidative stress and neuronal inflammation lead to the occurrence of autism. Autism is associated with reduced expression of the ZNF804A.

ZNF804A copy number variations (CNVs) have also been observed in individuals with autism. Zhang et al. (2019) found a close relationship between autism susceptibility and zinc finger protein804A (C2H2 type) in a study of autistic patients in Han Chinese population. zinc finger protein804A, a nuclear protein derived from C2H2 subtype, can interact with neuronal RNA splicing factors and RNA-binding proteins to promote gene expression. In neuronal cells, pre-mRNA processing can be regulated by ZNF804A through RNA-binding proteins which suggests ZNF804A may relate to autism spectrum disorder (ASD) (Chapman et al., 2019). As shown in Figure 6, autism was found to be associated with decreased expression of the ZNF804A allele—which is caused by a variant of rs10497655 in the promoter. This variation leads to the increase of T allele in rs10497655, strengthens the affinity between HSF2 (Heat shock transcription Factor 2) and rs10497655 in the promoter, and thus inhibits the expression of ZNF804A, leading to autism (Zhang et al., 2019). For the zinc finger protein ZNF804A, variation of intron rs7603001 is associated with speech disorders in autistic patients (Anitha et al., 2014).

ZNF292 and ZNF462 from C2H2 type subfamily are also related to ASD. Studies show that variants in ZNF292, which encodes a highly conserved zinc finger protein that is highly expressed in the developing human brain as a transcription factor, supporting its critical role in neural development, are associated with a spectrum of neurodevelopmental features including intellectual disability (ID), ASD, attention deficit and hyperactivity disorder (ADHD), among others (Mirzaa et al., 2020). Kruszka et al. (2019) describe a multiple congenital anomaly syndrome associated with haploinsufficiency of zinc finger protein 462 in which most patients have developmental delay (79%) and a minority have ASD (33%). Zinc finger and BTB domain containing 16 (ZBTB16) from C2H2 type subfamily plays the roles in the neural progenitor cell proliferation and neuronal differentiation during development, and associates with ASD and schizophrenia pathobiology (Usui et al., 2021). Another zinc finger protein associated with neural development is BAZ2B, which from PHD-type can interact with ISWI (imitation switch) and may play a role in transcriptional regulation (Jones et al., 2000; Oppikofer et al., 2017). BAZ2B haploinsufficiency is another reason for autism. Researches showed that BAZ2B haploinsufficiency plays an important role in neurodevelopmental disorder which may contribute to autism (Lalli et al., 2016; Scott et al., 2020). The above results all indicate that ZNF play important roles in autism, but the specific mechanism is still not clear, and many functions of ZNF have not been explored.

## Conclusion and Perspectives

In addition to the above ZNF, there are also ZNF associated with brain related diseases. Like zinc finger protein with KRAB and SCAN domains 3 (ZKSCAN3) from C2H2 type, downregulation of ZKSCAN3 is observed in aged human mesenchymal stem cells (hMSCs) and depletion of ZKSCAN3 accelerates senescence of these cells (Hu et al., 2020; Turelli et al., 2020). ZNF also play an important role in brain tumor disease (Balogh et al., 2020; Liang et al., 2020; Natsumeda et al., 2021; Okado, 2021). The AN1/A20 zinc finger domain containing protein 3 (ZFAND3) as a crucial driver of glioblastoma invasion. loss of ZFAND3 hampers the invasive capacity of GBM, whereas ZFAND3 overexpression increases motility in cells that were initially not invasive (Schuster et al., 2020). Zfp189, which encodes a previously unstudied zinc finger protein, is a novel antidepressant target, and overexpression of Zfp189 (C2H2 type) in prefrontal cortical neurons preferentially activates this network and promotes behavioral resilience (Lorsch et al., 2019). The accumulation ZNF746 (C2H2 type) and aminoacyl tRNA synthetase complex will result in Parkinson’s disease (Brahmachari et al., 2019). In the late stage of Parkinson’s disease, the depletion of dopaminergic neurons leads to the aggravation of oxidative stress in the brain and the corresponding increase of MT level, which plays a neuroprotective role and protects against the oxidative stress (Miyazaki,, Asanuma et al., 2007). Studies have shown that MT might be used as a new biomarker of PD (Sharma et al., 2013). The Morc family CW-type zinc finger 1 (Morc1) gene connects with early life stress and depression (Mundorf et al., 2021). Pathogenic mutation of ZNF711 (C2H2 type) leads to syndromic ID in children (Poeta et al., 2021). There are many ZNF that play a role in a variety of neuro-related diseases. For example, ZNF526 (C2H2 type) biallelic variants affect neurodevelopmental disorder, epileptic encephalopathy, bilateral cataracts and so on (Dentici et al., 2021). Our knowledge about ZNF so far indicate that they play an important role in the developing as well as the adult brain, and animal studies suggest that this network influence behaviors linked to psychiatric disorders. However, it is necessary to point out that a shortcoming of many of the zinc finger protein-studies is that they do not clearly discriminate structures, classifications, functions of ZNF, making it difficult to attribute the observed phenotypes to zinc finger protein-dysregulation. In addition, the underlying reasons for how ZNF bind RNA, DNA or protein in neuron, astrocytes, microglia, oligodendrocyte, and other cells of the brain remain unresolved but most likely include several parallel mechanisms. Recent structural studies of ZNF have shed new insights into their extraordinary diversity of structure and function. It is chastening to realize, however, that of the large number of putative zinc finger motifs that have been identified, only a handful have been characterized structurally.

According to this model, transposable elements of zinc finger protein sequences have been domesticated and now play an important role in fine-tuning transcriptional levels of numerous protein coding genes. Considering the fact that thousands of ZNF are expressed in the brain that in turn bind to thousands of transposable elements, it is likely that many protein coding genes are under the influence of zinc finger protein network. This is a very attractive hypothesis since ZNF are very suitable to drive evolution and the presence of many primate- and human specific zinc finger protein may then implicate these elements in the evolution of the complex primate brain. Regulation of RNA metabolism is an important component of gene expression that facilitates the fine-tuning of transcript levels during physiological conditions and during the rapid and profound switch in global gene expression associated with inflammation and immune responses. Long-term dysregulation of RNA metabolism can often result in disease states, including inflammatory and autoimmune diseases. CCCH zinc finger proteins have emerged as important regulators of multiple facets of RNA metabolism and immune responses, with promising therapeutic potential.

We favor a model where all these mechanisms are at play at once. However, the experimental data that support these mechanisms is still sparse. Future studies of zinc finger protein controlling regulation in the brain need to resolve the underlying mechanisms, in other words, they should determine if transposable elements regulation contributes to host fitness and if dysregulation of these networks contribute to human brain disorders. Addressing these questions will likely provide detail evidence that ZNF play a key role in the control of transcriptional networks in both the healthy and diseased brain.

## Author Contributions

SYB, YHL, and YSL designed the research and collected the materials. SQ and HMW wrote and amended the manuscript. All authors contributed to the article and approved the submitted version.

## Conflict of Interest

The authors declare that the research was conducted in the absence of any commercial or financial relationships that could be construed as a potential conflict of interest.

## Publisher’s Note

All claims expressed in this article are solely those of the authors and do not necessarily represent those of their affiliated organizations, or those of the publisher, the editors and the reviewers. Any product that may be evaluated in this article, or claim that may be made by its manufacturer, is not guaranteed or endorsed by the publisher.

## Abbreviations

AD: Alzheimer’s disease
AMPA: α-amino-3-hydroxy-5-methyl-4-isoxazole-propionic acid
BAZ2B: the bromodomain adjacent to zinc finger 2B gene
CRP: C-reactive protein
Cys: cysteine
EBF: early B-cell factor
ERK5: extracellular signal-regulated kinase 5
His: histidine
KIL4: Krüppel-like factor 4
MCPIP1: monocyte chemotactic protein-induced protein-1
MT: metallothionein
MZF1: myeloid zinc finger 1
NMDAR: N-Methyl-D-aspartate receptor
PPARγ: peroxisome proliferator-activated receptor gamma
ROS: reactive oxygen species
SCZ: Schizophrenia
SET: SE translocation
SGZ: sub-granular zone
TAZ2 domain: the second transcription adaptor putative zinc finger domain
TJs: tight junctions
TRIM28: tripartite motif-containing 28
ZBTB5: zinc finger and BTB domain-containing 5
ZFX: X-chromosome-coupled zinc finger protein
ZHNF: early hematopoietic zinc finger protein
ZKSCAN3: zinc finger protein with KRAB and SCAN domains 3
ZNF: zinc finger proteins.
